# The Baby Care Scale: A Psychometric Study With Fathers During Pregnancy and the Postpartum Period

**DOI:** 10.3389/fpsyg.2021.751330

**Published:** 2022-01-17

**Authors:** Tiago Miguel Pinto, Rui Nunes-Costa, Bárbara Figueiredo

**Affiliations:** Psychology Research Centre, School of Psychology, University of Minho, Braga, Portugal

**Keywords:** Baby Care Scale, psychometric characteristics, fathers, involvement in infant care, anxiety and depressive symptoms, father–infant emotional involvement

## Abstract

The Baby Care Scale (BCS) was designed to assess the involvement of father in infant care during pregnancy and the postpartum period. This study aimed to examine the psychometric characteristics of the BCS – antenatal (BCS-AN) and BCS – postnatal (BCS-PN) versions. A sample of 100 primiparous fathers completed the BCS-AN and/or the BCS-PN and self-reported the measures of anxiety and depressive symptoms and of father–infant emotional involvement during pregnancy and the postpartum period, respectively. Good internal consistency was found for both the BCS-AN and the BCS-PN. A two-factor model was found for both versions of the instrument: (1) household tasks and (2) infant care tasks. The BCS-AN and BCS-PN subscales revealed good internal consistency. Higher scores on the BCS-AN predicted higher scores on the BCS-PN. Significant associations were found among the BCS (BCS-AN and BCS-PN), depressive and anxiety symptoms, and father–infant emotional involvement, revealing good criterion validity. This study suggested that both the BCS-AN and the BCS-PN are reliable multidimensional self-report measures that assess the involvement of father in infant care during pregnancy and the postpartum period.

## Introduction

During the past several decades, major changes occurred in family structures. Thus, the role of men as fathers has changed, and an increase in their involvement in infant caregiving has been observed (e.g., Parke, 2004). Although the knowledge on father–infant relationships has increased (Lamb, 1981, 2013), validated measures to assess the involvement of father in infant care are scarce, which limits research in this field.

Literature has consistently demonstrated an association between the quality of involvement of father in infant care and infant physical and mental development (e.g., Lamb, 2010). The impact of involvement of father in infant care on infant development may occur either directly, through their behaviors, or indirectly, through its impact on the perceived support of mother and consequently on their mental health and parenting (Parke, 1979; Belsky, 1984). Indirect support can then be operationalized in the support of father to the care provided by the mother or through shared responsibilities in caring for the child and sharing domestic tasks. Moreover, the involvement of father in infant care and the household is associated with a greater subjective well-being of the mother (e.g., Lennon et al., 1991) and a better quality of interaction with the infant (e.g., Brunelli et al., 1995; Bögels and Brechman-Toussaint, 2006).

According to the developmental perspective (Cowan, 1991), fatherhood should not be defined exclusively by the moment of birth. The care provided to the offspring begins during pregnancy and is concomitant with a wide range of developmental tasks to be performed by both the mother and the father (Figueiredo, 2013). Activities performed by the father, namely, being present in the ultrasounds or taking care of the infant arrival, are major predictors of his emotional involvement with the infant during pregnancy (e.g., Samorinha et al., 2009). Likewise, the higher levels of support received by the father, both emotional and instrumental, are associated with higher subjective well-being of the pregnant woman and the acceptance of her pregnancy (Cutrona, 1996; Kroelinger and Oths, 2000; Rini et al., 2006).

The Baby Care Scale (BCS; Figueiredo, 1997) was specifically designed to assess the involvement of father in infant care during pregnancy and the first-year postpartum. In its original version, this scale was filled in by the mother to assess different tasks of infant care (Figueiredo, 1997). The items assess the frequency of involvement of father in household and infant care tasks. Although the original version that presented good psychometric characteristics was completed by the mothers (Figueiredo, 1997), this study used a small sample and, to the best of our knowledge, the psychometric characteristics of the BCS were not yet tested in fathers. This study aimed to examine the psychometric characteristics of the BCS – antenatal (BCS-AN) and BCS – postnatal (BCS-PN) versions in fathers.

Literature highlights the impact of involvement of father in infant care during pregnancy and the postpartum period on infant development (Ramchandani et al., 2011; Gutierrez-Galve et al., 2015). Moreover, literature also provides evidence on the negative impact of adjustment problems of father to the transition to parenthood on their own parenting (Vismara et al., 2016; Rollè et al., 2017). Altogether, these studies provide evidence that perinatal screenings and interventions should target both mothers and fathers early in the perinatal period. Analyzing the psychometric characteristics of the BCS-AN and the BCS-PN in fathers could be a major contribute to the field on adjustment of father to the transition to parenthood. Both the BCS-AN and the BCS-PN could be useful tools to assess the involvement of father in infant care and identify fathers with low involvement in infant care during pregnancy and/or the postpartum period.

## Materials and Methods

### Participants

The sample comprised 100 primiparous fathers who completed the BCS-AN and the BCS-PN. Participants were between the ages of 20 and 45 years (*M* = 31.34, SD = 4.38). Nearly all the participants were Portuguese (97.9%) and Caucasian (82.1%), employed (89.5%), married or cohabiting (91.4%), and lived only with the partner (75.8%). Of these 100 participants, 85 (85%) completed the BCS-AN. Of these 85 participants, 35.3% (*n* = 30) did not continue the study. Thus, 55 fathers completed both the BCS-AN and the BCS-PN. In addition, the BCS-PN was also completed by 15 new participants specifically recruited in this period (*n* = 70) (see Table 1).

**TABLE 1:** Sociodemographic characteristics of participants.

		BCS-AN^1^	BCS-PN^2^	BCS-AN and PN^3^	Total
		*n* = 85	*n* = 70	*n* = 55	*N* = 100
		%	%	%	%
Age (years)	20–29	35.3	25.7	30.9	31.6
	30–39	62.4	70.0	65.5	65.3
	>40	2.4	4.3	3.6	3.2
Occupational status	Employed	88.2	91.4	90.9	89.5
	Unemployed	9.4	7.1	7.3	8.4
	Student	2.4	1.4	1.8	2.1
Education (in years)	<9	9.4	4.3	5.5	8.4
	9–12	55.3	54.3	63.6	50.5
	>12	35.3	58.3	30.9	41.1
Marital status	Married	64.7	67.1	65.5	63.3
	Cohabiting	27.1	25.7	25.5	28.1
	Single	8.2	7.1	9.1	26.3
Mode of conception	Spontaneous	96.5	94.3	96.4	94.7
	Medically assisted	3.5	5.7	3.6	5.3

*^1^Completed the BCS-AN.*

*^2^Completed the BCS-PN.*

*^3^Completed both the BCS-AN and the BCS-PN. BCS-AN, Baby Care Scale – antenatal version; BCS-PN, Baby Care Scale – postnatal version.*

### Procedure

This study was approved by the Ethics Committees of all institutions involved and complied with the standards and recommendations provided by the Declaration of Helsinki. Participation was voluntary, and all participants were informed about aims and procedures of the study and signed an informed consent form. Participants were recruited in two Portuguese Health Services (one private and one public) during the first trimester of pregnancy or during the first-year postpartum. Inclusion criteria were being able to read and write European Portuguese, lived in Portugal for the past 10 years, be primiparous fathers of a single pregnancy, and have no gestational problems. Participants were randomly recruited between October 2013 and March 2015, after the first ultrasound (between 8 and 14 weeks of gestation) or in the postpartum period (between 1 and 12 months postpartum). Fathers completed the BCS-AN and/or the BCS-PN online and self-reported the measures of anxiety and depressive symptoms and of father–infant emotional involvement during pregnancy and the postpartum period, respectively.

### Measures

#### Sociodemographic Questionnaire

A sociodemographic questionnaire (Figueiredo et al., 2009) was used to collect the sociodemographic information of father (e.g., age, ethnicity, religion, occupational status, and educational level).

#### Baby Care Scale – Antenatal and Baby Care Scale – Postnatal Versions

The BCS (Figueiredo, 1997) was designed to assess the involvement of father in infant care during the first-year postpartum. The scale consists of 14 items scored on a 4-point Likert-type scale (from 0 to 3) that assessed the frequency of tasks of father related to the infant care. The BCS includes items that assessed the involvement of father in household tasks (e.g., preparing meals, cleaning the kitchen, and shopping) and related to the care directly provided to the infant (e.g., feeding the infant, bathing the infant, and changing diapers when wet). The total score of BCS ranges between 0 and 42, with higher scores indicating higher involvement of father in infant care. The original version was developed by Figueiredo (1997), presenting good internal consistency (Cronbach’s alpha = 0.82).

The BCS-PN was adapted to be used with fathers during pregnancy (BCS-AN), maintaining the overall structure of the BCS-PN. Although the items that assessed the involvement of father in the household tasks remained in the prenatal version, the content of the items that assessed the involvement of father in infant care tasks was modified to assess the care provided to the infant and the pregnant mother (Parke, 1979; Belsky, 1984) and involvement of father in the preparations of the infant arrival (e.g., Colman and Colman, 1994).

#### Baby Care Scale – Antenatal and Baby Care Scale – Postnatal Criterion Validity

Measures of anxiety and depression symptoms and of father–infant emotional involvement were used to assess criterion validity for both BCS-AN and BCS-PN. The Edinburgh Postnatal Depression Scale (EPDS; Cox et al., 1987) was used to assess depressive symptoms during pregnancy and the postpartum period. The EPDS is a 10-item self-report scale scored on a 4-point Likert-type scale, designed to assess postpartum depression. This measure assesses the intensity of depressive symptoms within the previous 7 days and has been used in several studies with men during pregnancy and the postpartum period (e.g., Figueiredo and Conde, 2011; Parfitt and Ayers, 2014). The EPDS Portuguese version showed good internal consistency during pregnancy and the postpartum period (e.g., Figueiredo and Conde, 2011). In this sample, Cronbach’s alpha coefficient was 0.80 during pregnancy and 0.77 during the postpartum period.

The State-Trait Anxiety Inventory (STAI; Spielberger et al., 1983) is a self-report scale comprised by two subscales, namely, the state anxiety and the trait anxiety, each containing 20 items scored on a 4-point Likert-type scale. The State Anxiety Inventory (STAI-S) that measures the temporary condition of “state anxiety” (anxiety in a specific situation) was used to assess anxiety symptoms during pregnancy and the postpartum period. Several studies have used this measure with men during pregnancy and the postpartum period (e.g., Figueiredo and Conde, 2011). The STAI-S Portuguese version showed good internal consistency (α = 0.88; Biaggio et al., 1976). In this sample, Cronbach’s alpha coefficient was 0.88 during pregnancy and 0.92 during the postpartum period.

The Portuguese version of the Mother-Infant Bonding Scale (MIBS; Figueiredo, 2013) was used to assess father–infant emotional involvement. The items are organized into three subscales, with a total of 11 self-report items, rated on a 4-point Likert-type scale. The “Positive Bonding” subscale consists of three items (i.e., Affectionate, Protective, and Happy) and measures positive emotional involvement; the “Negative Bonding” subscale consists of six items (i.e., Angry, Aggressive, Sad, Resentful, Disgusted, and Disillusioned) and assesses negative emotional involvement; the subscale “Bonding not clear” consists of two items (i.e., Afraid and Neutral and No Feelings) and signals the presence of emotions not clearly related to the emotional involvement of parents with the infant. This scale has been used both during pregnancy and the postpartum period (e.g., Samorinha et al., 2009; Brandão and Figueiredo, 2012). The MIBS showed good internal consistency (α = 0.71; Figueiredo and Costa, 2009). In this sample, Cronbach’s alpha coefficient was 0.72 during pregnancy and 0.78 during the postpartum period.

### Data Analysis

The analysis of the psychometric characteristics of both the BCS-AN and the BCS-PN included analysis of (1) factor structure and internal consistency and (2) criterion validity and predictive validity. The analysis of (1) factor structure and internal consistency included factor analysis in principal components, with orthogonal rotation using the varimax method, as well as a confirmatory factor analysis for each scale. To ensure that each item represented the construct underlying the factor, a minimum factor loading of 0.30 was considered. The suitability of the items to factor analysis was examined by the Kaiser–Meyer–Olkin (KMO) measure and Bartlett’s sphericity test (AIC). The internal consistency of the BCS-AN and the BCS-PN was analyzed using Cronbach’s alpha coefficient and item-total and mean-item correlations (MICs). Values of Cronbach’s alpha ≥0.70, item-total correlations (ITCs) ≥0.30, and mean inter-item correlations >0.15 all indicate good level of internal consistency (Nunnally and Bernstein, 1994; Field, 2005). Pearson’s correlations were also performed to analyze the intercorrelations of both scales. To analyze (2) criterion validity, Pearson’s correlations were performed between the BCS-AN and the BCS-PN (total scale and subscales) and measures of anxiety (STAI-S) and depressive (EPDS) symptoms and measures of father–infant emotional involvement (MIBS). To analyze (2) the predictive validity, participants who completed both versions of the ECPB (prenatal and postnatal) were also included in a linear regression analysis to examine the predictive value of the BCS-AN scores on the BCS-PN scores.

## Results

### Baby Care Scale – Antenatal and Baby Care Scale – Postnatal Factor Structure

Regarding the BCS-AN, the adequacy of the factor analysis to the items, by verifying the existence of significant correlations between them, was confirmed by the KMO measure (KMO = 0.73) and Bartlett’s sphericity test (QQ = 434.6, df = 91, *p* < 0.001). The results obtained in the factor analysis showed as the best solution two factors that explained, in its entirety, 46.82% of the variance. The inflection point of the Cattell (1996) supported a two-factor solution. The first factor contains five items related to the involvement of father in household activities and contributes 25.1% of the total explained variance.

Table 2 shows the results of saturations by item as well as the ITCs, which ranged between 0.27 and 0.48, and the average inter-item correlation, which were 0.15 greater on each subscale. The Cronbach’s alpha found was 0.81.

**TABLE 2:** BCS-AN exploratory factor analysis.

	Factor loadings		ITC	α IID	*C*	*M*	SD
Item	Factor 1	Factor 2					
Household tasks subscale (α = 0.76)			MIC = 0.39				

1. Prepare meals	0.77		0.48	0.79	0.59	1.80	0.94
2. Clean the kitchen	0.67		0.36	0.80	0.45	2.26	0.90
3. Perform household tasks (e.g., vacuuming, washing)	0.80		0.46	0.79	0.64	1.69	0.90
4. Go shopping	0.69		0.48	0.79	0.49	1.74	0.71
5. Make small arrangements at home	0.55		0.27	0.81	0.31	1.48	0.77

Infant care tasks subscale (α = 0.79)			MIC = 0.33				

6. Touch your partner’s belly		0.60	0.45	0.79	0.39	2.72	0.65
7. Talk to your partner’s belly		0.58	0.68	0.77	0.60	1.89	0.98
8. Refers to the baby by his/her name (name you and your partner decided to give)		0.36	0.46	0.80	0.32	1.55	1.27
9. Talk about the baby		0.62	0.44	0.80	0.40	2.81	0.55
10. Participate in the purchase of essential goods for the baby (e.g., crib, clothes)		0.57	0.46	0.79	0.38	2.26	0.85
11. Imagine your baby		0.63	0.39	0.80	0.40	2.19	0.89
12. Accompany your partner to the obstetrician		0.62	0.35	0.80	0.45	2.66	0.73
13. It is present in the baby’s ultrasounds		0.70	0.42	0.80	0.50	2.72	0.68
14. Think about how your baby is doing		0.79	0.41	0.80	0.64	2.66	0.63

BCS-AN total (α = 0.81)						30.44	6.23

*C, communalities; ITC, item-total correlation; MIC, mean-item correlation; IID, if item deleted; M, mean; SD, standard deviation; BCS-AN, Baby Care Scale – antenatal version.*

Regarding the BCS-PN, the KMO measure (KMO = 0.74) and Bartlett’s sphericity test (QQ = 390.0, df = 78, *p* < 0.001) revealed the adequacy of factor analysis. The best solution revealed a two-factor structure that explains 45.03% of the variance. Again, the inflection point of the Cattell (1996) supports a two-factor solution. The first factor contains 8 items related to the care provided directly to the baby and contributes 21.2% of the total explained variance. The second factor contains the remaining 6 items related to the involvement of father in domestic activities and contributes 19.9% of the total explained variance.

The results of saturations by item as well as the ITCs, which varied between 0.26 and 0.62, are shown in Table 3. Also, as in the BCS-AN, inter-item correlation means were 0.15 higher on each of the subscales. The Cronbach’s alpha for the BCS-PN was 0.82.

**TABLE 3:** BCS-PN exploratory factor analysis.

	Factor loadings		ITC	α IID	*C*	*M*	SD
Item	Factor 1	Factor 2					
Household tasks subscale (α = 0.74)			MCI = 0.29				

1. Prepare meals		0.75	0.40	0.81	0.57	1.96	0.91
2. Clean up the kitchen		0.71	0.48	0.80	0.54	2.17	0.83
3. Perform household tasks (e.g., vacuuming, washing)		0.75	0.49	0.80	0.58	1.66	0.74
4. Go shopping		0.67	0.37	0.81	0.44	1.83	0.56
5. Make small arrangements at home		0.46	0.32	0.81	0.24	1.44	0.65
13. Take care of the house and the baby		0.43	0.46	0.80	0.32	2.37	0.75

Infant care tasks subscale (α = 0.78)			MCI = 0.32				

6. Feed the baby	0.80		0.51	0.80	0.64	2.21	0.86
7. Bathe the baby	0.31		0.26	0.82	0.13	2.27	0.82
8. Change diapers even when dirty with poop	0.62		0.56	0.80	0.48	2.54	0.85
9. Put the baby to sleep	0.90		0.63	0.80	0.81	2.54	0.77
10. Change diapers even when wet	0.90		0.62	0.80	0.80	2.54	0.77
11. Get up at night to take care of the baby	0.55		0.41	0.81	0.39	2.21	0.88
12. Take the baby for a walk	0.51		0.54	0.80	0.33	2.19	0.64
14. Play with the baby	0.15		0.13	0.82	0.04	2.79	0.59

BCS-PN total (α = 0.82)						30.39	5.83

*C, communalities; ITC, item-total correlation; MIC, mean-item correlation; IID, if item deleted; BCS-PN, Baby Care Scale – postnatal version.*

Pearson’s correlations revealed that the BCS-AN total scale was significantly correlated with both subscales: household tasks (*r* = 0.72, *p* < 0.001) and the infant care tasks (*r* = 0.89, *p* < 0.001). Significant correlations were also found between the two BCS-AN subscales (*r* = 0.32, *p* = 0.003). Pearson’s correlations also revealed that the BCS-PN total scale was significantly correlated with both subscales: household tasks (*r* = 0.79, *p* < 0.001) and the infant care tasks (*r* = 0.89, *p* < 0.001). Significant correlations were also found between the two BCS-PN subscales (*r* = 0.42, *p* < 0.001) (see Table 4).

**TABLE 4:** BCS-AN and BCS-PN intercorrelations.

	BCS-AN	Household tasks	Infant care tasks
BCS-AN	1.00		
Household tasks	0.72***	1.00	
Infant care tasks	0.89***	0.32**	1.00

	**BCS-PN**	**Household tasks**	**Infant care tasks**

BCS-PN	1.00		
Household tasks	0.79***	1.00	
Infant care tasks	0.89***	0.42***	1.00

BCS-AN, Baby Care Scale – antenatal version; BCS-PN, Baby Care Scale – postnatal version. **p < 0.01 and ***p < 0.001.

### Baby Care Scale – Antenatal and Baby Care Scale – Postnatal Criterion and Predictive Validity

Pearson’s correlations revealed significant negative correlations between anxiety and depressive symptoms during pregnancy and the BCS-AN total scale and subscales. Likewise, significant negative correlations between anxiety and depressive symptoms during the postpartum period and the BCS-PN total scale and subscales were found (see Table 5).

**TABLE 5:** BCS-AN and BCS-PN criterion validity: correlations with anxiety and depressive symptoms, and father–infant emotional involvement during pregnancy and the postpartum period, respectively.

Pregnancy	BCS-AN	Household tasks	Infant care tasks
Anxiety symptoms STAI-S	−0.3**	−0.28*	−0.27*
Depressive symptoms EPDS	−0.48***	−0.40***	−0.46***
Total MIBS	0.28**	0.02	0.36***
Positive MIBS	0.40***	0.06	0.50***
Negative MIBS	0.08	0.07	0.07
Not clear MIBS	–0.09	–0.04	–0.09

**Postpartum**	**BCS-PN**	**Household tasks**	**Infant care tasks**

Anxiety symptoms STAI-S	−0.29*	−0.21*	−0.24*
Depressive symptoms EPDS	−0.41***	−0.38***	−0.44***
Total MIBS	0.098	–0.019	0.159
Positive MIBS	0.274*	0.178	0.272*
Negative MIBS	0.009	0.119	–0.076
Not clear MIBS	–0.006	0.065	–0.057

BCS-AN, Baby Care Scale – antenatal version; BCS-PN, Baby Care Scale – postnatal version. *p < 0.05; **p < 0.01; ***p < 0.001.

Pearson’s correlations revealed significant positive correlations between the MIBS total score during pregnancy and the BCS-AN total score (*r* = 0.28, *p* = 0.010), as well as with the infant care tasks subscale (*r* = 0.36, *p* = 0.001). The positive MIBS subscale was positively correlated with the BCS-AN total score (*r* = 0.40, *p* < 0.001) and the infant care tasks subscale (*r* = 0.51, *p* < 0.001).

Significant positive correlations were also found between the BCS-PN total score and the MIBS total score and positive subscale (*r* = 0.27, *p* = 0.022), as well as with the infant care tasks subscale (*r* = 0.27, *p* = 0.023) (see Table 5).

A linear regression analysis performed with participants who completed both versions of the ECPB also revealed the good predictive validity for the BCS-AN [adjusted *R*^2^ = 0.34, *F*(1,53) = 28.74, *p* < 0.001]. Higher scores on the BCS-AN significantly predicted higher scores on the BCS-PN (β = 0.60, *t* = 5.36, *p* < 0.001).

## Discussion

This study provided evidence that the BCS has good psychometric characteristics in fathers, both in the BCS-AN and BCS-PN versions. The BCS-AN and the BCS-PN showed good internal consistency, both considering the Cronbach’s alpha values (α = 0.81 for the BCS-AN and α = 0.82 for the BCS-PN) and the ITCs with values greater than 0.30 and mean of inter-item correlations greater than 0.15 (Field, 2005). A two-factor model was found for both the BCS-AN and the BCS-PN. The first factor included items related to the care provided by the father directly to the infant (infant care tasks), while the second factor included all the items related to the household tasks provided by the father (household tasks). All subscales of both the BCS-AN and the BCS-PN showed good internal consistency (with α ranging between 0.74 and 0.82). Moreover, a significant prediction of the BCS-AN scores was found on the BCS-PN scores. Results provided evidence that fathers who provide more care to the infant during pregnancy can be those who provide more care to the infant during the postpartum period.

Although item 14 presented a factor loading and an ITC values below the recommended (Field, 2005), it was maintained. The maintenance of this item did not interfere with the internal consistency of the scale and the number of items in the original scale (Figueiredo, 1997) was preserved. However, it is recommended that future studies should further analyze the internal consistency of the item 14, namely, using larger samples of fathers, considering the influence of the sample size on internal consistency (Field, 2005).

Regarding the BCS-AN and BCS-PN criterion validity, significant medium-sized correlations were obtained between the BCS-AN and BCS-PN and measures of anxiety and depressive symptoms and measures of father–infant emotional involvement. Specifically, significant associations were found between mental health problems of father and the frequency of care provided by the father to the infant. These results are in line with previous studies, suggesting the negative impact of mental health problems of father on their own parenting (e.g., Vismara et al., 2016; Rollè et al., 2017). Likewise, significant associations were found between the frequency of care provided by the father to the infant and the quality of the emotional involvement of father with the infant. Higher values in the MIBS total scale and in the positive subscale were associated with higher frequency of care from the father to the infant and to the partner, both during pregnancy and the postpartum period (Figueiredo and Costa, 2009). Previous studies have shown that the quality of bonding established with the infant during pregnancy is associated with the quality of care provided by the father (e.g., Wiberg et al., 1989).

A significant prediction of the BCS-AN scores was found on the BCS-PN scores. Results provided evidence that fathers who provide more care to the infant during pregnancy can be those who provide more care to the infant during the postpartum period. Fathers with low involvement in infant care can be identified early during pregnancy.

### Limitations

The voluntary nature of participation in this study may work as a bias of the results found, as those who participated in this study may be those who are already more involved in providing care to the infant. A larger and a more social culturally diverse sample of fathers would have allowed to conduct more robust psychometric analyses on the BCS-AN and BCS-PN, namely, a confirmatory factor analysis.

### Implications for Practice and Research

Literature provided evidence that perinatal screenings and interventions should target both mothers and father early in the perinatal period (Gutierrez-Galve et al., 2015; Vismara et al., 2016; Rollè et al., 2017). The BCS-AN and the BCS-PN may be reliable self-report measures to assess the involvement of father in infant care and may allow perinatal practitioners to screen fathers with low involvement in infant care during pregnancy and/or the postpartum period. The use of BCS as part of routine perinatal care appointments may provide an easy and valid strategy to identify fathers with low involvement in infant care and to provide information on those who could benefit from perinatal psychological counseling.

For researchers, both versions of the BCS may be useful measures in longitudinal studies on the involvement of father in infant care during pregnancy and the postpartum period. Both versions of the BCS could also be useful tools to assess the effectiveness of new interventions aiming to promote positive parenting and/or to prevent parenting difficulties in fathers during the perinatal period. This study provided preliminary evidence on the psychometric properties of the BCS in a community sample of fathers during the transition to parenthood. Future studies might assess other psychometric properties of both versions of the BCS using larger and social culturally diverse samples of fathers during the perinatal period.

## Conclusion

This study suggested that the BCS-AN and the BCS-PN are reliable multidimensional self-reported measures to assess the involvement of father in infant care during pregnancy and the postpartum period.

## Data Availability Statement

The raw data supporting the conclusions of this article will be made available by the authors, without undue reservation.

## Ethics Statement

The studies involving human participants were reviewed and approved by the University of Minho. The patients/participants provided their written informed consent to participate in this study.

## Author Contributions

All authors participated in the study design, undertook the statistical analysis, interpreted the results, and wrote the first draft of the manuscript. TP and RN-C collected the data. All authors contributed to and have approved the final manuscript.

## Conflict of Interest

The authors declare that the research was conducted in the absence of any commercial or financial relationships that could be construed as a potential conflict of interest.

## Publisher’s Note

All claims expressed in this article are solely those of the authors and do not necessarily represent those of their affiliated organizations, or those of the publisher, the editors and the reviewers. Any product that may be evaluated in this article, or claim that may be made by its manufacturer, is not guaranteed or endorsed by the publisher.

## References

[B1] Belsky J. (1984). The determinants of parenting: a process model. *Child Dev.* 55 83–96. 10.2307/1129836 6705636

[B2] Biaggio A. M. Natalicio L. Spielberger C. D. (1976). “The development and validation of an experimental Portuguese form of the State-Trait Anxiety Inventory,” in *Cross-Cultural Research on Anxiety*, eds Spielberger C. D. Dias-Guerrero R. (Washington, DC: Hemisphere), 29–40.

[B3] Bögels S. M. Brechman-Toussaint M. L. (2006). Family issues in child anxiety: attachment, family functioning, parental rearing and beliefs. *Clin. Psychol. Rev.* 26 834–856. 10.1016/j.cpr.2007.07.011 16473441

[B4] Brandão S. Figueiredo B. (2012). Fathers’ emotional involvement with the neonate: impact of the umbilical cord cutting experience. *J. Adv. Nurs.* 68 2730–2739. 10.1111/j.1365-2648.2012.05978.x 22458831

[B5] Brunelli S. A. Wasserman G. A. Rauh V. A. Alvarado L. E. Caraballo L. R. (1995). Mothers’ reports of paternal support: associations with maternal child-rearing attitudes. *Merrill Palmer Q.* 41 152–171.

[B6] Cattell R. B. (1966). The scree test for the number of factors. *Multivar*. *Behav. Res.* 1, 245–276. 10.1207/s15327906mbr0102_10 26828106

[B7] Colman L. L. Colman A. D. (1994). *Pregnancy: The Psychological Experience.* Lisbon: Colibri Editions.

[B8] Cowan P. A. (1991). “Individual and family life transitions: a proposal for a new definition,” in *Family Transitions*, eds Cowan P. A. Hetherington M. (Hillsdale, NJ: Erlbaum Association), 3–26.

[B9] Cox J. L. Holden J. M. Sagovsky R. (1987). Detection of Postnatal depression: development of the 10-item Edinburgh Postnatal Depression Scale. *Br. J. Psychiatry* 150, 782–786. 10.1192/bjp.150.6.782 3651732

[B10] Cutrona C. E. (1996). “Social support as a determinant of marital quality: the interplay of negative and supportive behaviors,” in *Handbook of Social Support and the Family*, eds Pierce G. R. Sarason B. R. Sarason I. G. (New York, NY: Plenum Press). 10.3109/01612849009014544

[B14] Field A. (2005). *Discovering Statistics using SPSS.* London: SAGE Publications.

[B15] Figueiredo B. (1997). *Postpartum Depression, Mother-Infant Interaction and Child Development*. Unpublished Doctoral thesis, Clinical Psychology. Braga: University of Minho.

[B16] Figueiredo B. (2013). *Mothers and Fathers. Emotional Involvement With the Baby*, 1 Edn. Braga: Psyquilibriums.

[B12] Figueiredo B. Costa R. (2009). Mother’s stress, mood and emotional involvement with the infant: 3 months before and 3 months after childbirth. *Arch. Womens Ment. Health* 12, 143–153. 19259772 10.1007/s00737-009-0059-4

[B11] Figueiredo B. Conde A. (2011). Anxiety and depression symptoms in women and men from early pregnancy to 3-months postpartum: parity differences and effects. *J. Affect. Disord.* 132, 146–157. 10.1016/j.jad.2011.02.007 21420178

[B13] Figueiredo B. Teixeira C. Conde A. Pinto A. Sarmento P. (2009). Utentes da consulta externa de ginecologia/obstetrícia da Maternidade Júlio Dinis [Patients of the gynecologic/obstetric outpatient unit of Júlio Dinis maternity]. *Rev. Port. Psicol.* 41, 45–64.

[B17] Gutierrez-Galve L. Stein A. Hanington L. Heron J. Ramchandani P. (2015). Paternal depression in the postnatal period and child development: mediators and moderators. *Pediatrics* 135 e339–e347. 10.1542/peds.2014-2411 25560437

[B18] Kroelinger C. D. Oths K. S. (2000). Partner support and pregnancy wantedness. *Birth* 27 112–119. 10.1046/j.1523-536x.2000.00112.x 11251489

[B19] Lamb M. (2010). “How do father influence children’s development? Let me count the ways,” in *The Role of the Father in Child Development*, 5th Edn. ed. Lamb M. (Hoboken, NJ: Wiley), 1–26.

[B20] Lamb M. E. (1981). “Fathers and child development: an integrative overview,” in *The Role of the Father in Child Development*, ed. Lamb M. E. (New York, NY: Wiley), 1–70.

[B21] Lamb M. E. (2013). *The Father’s Role: Cross Cultural Perspectives.* Abingdon: Routledge.

[B22] Lennon M. C. Wasserman G. A. Allen R. (1991). Infant care and wives’ depressive symptoms. *Women Health* 17 1–23. 10.1300/J013v17n02_01 1871985

[B23] Nunnally J. C. Bernstein I. H. (1994). The assessment of reliability. *Psychom. Theory* 3 248–292.

[B24] Parfitt Y. Ayers S. (2014). Transition to parenthood and mental health in first-time parents. *Infant Ment. Health J.* 35, 263–273. 10.1002/imhj.21443 25798480

[B25] Parke R. D. (1979). “Perspectives on father-infant interaction,” in *Handbook of Infant Development*, ed. Osofsky J. D. (New York, NY: Wiley), 549–591.

[B26] Parke R. D. (2004). Fathers, families, and the future: a plethora of plausible predictions. *Merrill Palmer Q.* 50 456–470. 10.1353/mpq.2004.0033 34409987

[B27] Ramchandani P. G. Psychogiou L. Vlachos H. Iles J. Sethna V. Netsi E. (2011). Paternal depression: an examination of its links with father, child and family functioning in the postnatal period. *Depress. Anxiety* 28 471–477. 10.1002/da.20814 21506206 PMC3128925

[B28] Rini C. Schetter C. D. Hobel C. J. Glynn L. M. Sandman C. A. (2006). Effective social support: antecedents and consequences of partner support during pregnancy. *Pers. Relationsh.* 13 207–229. 10.1111/j.1475-6811.2006.00114.x

[B29] Rollè L. Prino L. E. Sechi C. Vismara L. Neri E. Polizzi C. (2017). Parenting stress, mental health, dyadic adjustment: a structural equation model. *Front. Psychol.* 8:839. 10.3389/fpsyg.2017.00839 28588541 PMC5441134

[B30] Samorinha C. Figueiredo B. Cruz J. M. (2009). Prenatal attachment and anxiety in mothers and fathers: impact of ultrasound in the 1st trimester of pregnancy. *Psicol. Saúde Doenças* 1 15–28.

[B31] Spielberger C. D. Gorsuch R. L. Lushene R. Vagg P. R. Jacobs G. A. (1983). *Manual for the State–Trait Anxiety Inventory: STAI (Form Y)*. Palo Alto, CA: Consulting Psychologists Press.

[B32] Vismara L. Rollè L. Agostini F. Sechi C. Fenaroli V. Molgora S. (2016). Perinatal parenting stress, anxiety, and depression outcomes in first-time mothers and fathers: a 3-to 6-months postpartum follow-up study. *Front. Psychol.* 7:938. 10.3389/fpsyg.2016.00938 27445906 PMC4919353

[B33] Wiberg B. Humble K. De Château P. (1989). Long-term effect on mother-infant behavior of extra contact during the first hour post partum. V. Follow-up at three years. *Scand. J. Public Health* 17 181–191. 10.1177/140349488901700209 2749204

